# Adherence to antihypertensive medication in renal denervation trials: new studies, old problems?

**DOI:** 10.1038/s41440-023-01389-6

**Published:** 2023-08-23

**Authors:** Lucas Lauder, Felix Mahfoud

**Affiliations:** Klinik für Innere Medizin III – Kardiologie, Angiologie und Internistische Intensivmedizin, Universitätskliniken des Saarlandes, Saarland University, Homburg, Germany

**Keywords:** Renal denervation, Device-based therapy, Hypertension

Hypertension is a leading cause of non-fatal and fatal cardiovascular complications [1]. Although pharmacological blood pressure-lowering reduces the risk of cardiovascular complications in patients without (primary prevention) and with (secondary prevention) previous cardiovascular events [2], disease awareness, and blood pressure control rates remain poor [3].

Renal denervation reduces blood pressure by modulating afferent and efferent renal sympathetic nerve activity. Recent sham-controlled trials have conclusively proven that ultrasound and radiofrequency renal denervation are both safe and effective in a broad range of patients with hypertension, including resistant hypertension [4]. Unlike antihypertensive medications, renal denervation lowers blood pressure continuously over 24 h, regardless of the patient’s adherence and independent of pharmacodynamics and -kinetics. Reducing night-time blood pressure, which is closely linked with the risk of coronary artery disease and heart failure, is particularly appealing [5].

Early renal denervation trials, most prominently the Symplicity HTN-3 trial, have sorely taught us that various measured and unmeasured confounders can affect the assessment of the blood pressure-lowering efficacy in clinical trials [4]. These include lifestyle modifications (especially during the COVID pandemic), the emergence of other health conditions, unblinding of patients and outcome assessors leading to performance bias, adjustments to concomitant antihypertensive pharmacotherapy to facilitate blood pressure control, and changes in adherence during the trial period [4]. To delineate the true effect of an intervention, it is fundamental to control for these factors. The *European Society of Cardiology (ESC) Council on Hypertension* and the *European Association of Percutaneous Coronary Interventions (EAPCI)* clinical consensus statement on renal denervation in the management of hypertension evaluated the scientific quality of renal denervation trials based on five methodological characteristics: (i) sham-controlled, multicenter trial design, (ii) adequate blinding of patients and outcome assessors, (iii) ambulatory BP change as the primary outcome, (iv) study completed as planned with outcome data available for nearly all randomized participants, and (v) use of second-generation RDN systems and procedural techniques [4].

The REQUIRE trial [6], whose post-hoc analysis is published in this issue of *The Journal*, was not judged to be of the highest possible quality because the treating physicians were not blinded to the patient’s treatment allocation, and there was no evaluation to ensure that the patients’ blinding was effectively maintained throughout the trial [4]. Additional shortcomings of the trial design are the lack of medication standardization, which likely increased blood pressure variability, and the absence of objective drug adherence measurements such as toxicological analysis of blood and/or urine [6]. Although the procedures in REQUIRE trial were performed using the Paradise ultrasound RDN catheter system, which was shown to lower blood pressure in three additional sham-controlled trials [7], the trial failed to meet its primary efficacy endpoint, the difference in change in 24-hour ambulatory systolic blood pressure at three months between the renal denervation and sham groups [6]. Compared with the sham group, RDN significantly reduced ambulatory systolic blood pressure at 1 month. However, the blood pressure also decreased in the sham group between one and three months, vanishing the initial between-group difference [6]. It is likely that the drop in blood pressure in the sham group is caused by an uneven intensification of antihypertensive pharmacotherapy in one group. Of note, outcomes were not different when patients with documented medication changes were excluded [6], but one has to keep in mind that changes in adherence and self-therapy by patients could not be excluded.

In this issue of *The Journal*, the authors report the results of a post-hoc analysis using urine samples of patients included in Japan to assess drug adherence toxicologically [8]. The key findings of this post-hoc analysis are that (i) drug adherence is appallingly poor in patients with (resistant) hypertension, as only about half of the patients (55%) showed good adherence (defined as a drug detection rate ≥ 75%) at baseline, (ii) drug adherence is worse in patients with a high medication burden, and (iii) renal denervation significantly lowered blood pressure compared with sham in patients with good adherence at baseline (−10.1 ± 13.3 mmHg versus −1.9 ± 15.3 mmHg) [8]. The most important limitation of this post-hoc analysis is that less than half of the patients enrolled in REQUIRE were included (58 of 136 patients) [8].

This post-hoc analysis is both timely and reassuring. Renal denervation effectively lowered blood pressure after excluding non-adherent patients, and the blood pressure reduction was consistent with previous trials [4]. Renal denervation is an adjunct treatment option in uncontrolled resistant hypertension and has recently been also recommended by current guidelines [4, 9]. Moreover, this analysis reminds us that non-adherence is a frequent cause of uncontrolled and pseudo-resistant hypertension, irrespective of geography and race, resulting in poor clinical outcomes and high healthcare costs [10]. Treatments should be simplified (preferably based on single-pill combinations) and tailored to the patient’s wants and needs to improve adherence. If a patient remains non-adherent and prefers an interventional treatment, renal denervation is a viable adjunct treatment option to facilitate blood pressure control [4].

The authors should be congratulated for publishing this analysis, which undoubtedly sheds light on the potential unexpected outcome of the REQUIRE trial. This analysis provides interesting information for clinical trialists and underscores that the addition of a sham procedure does not eliminate other sources of bias, such as variations in treatment score and dosages prescribed by the physicians and adherence to treatment by the patients [4].
